# P-1556. IFITM3 Genetic Variant rs34481144 and Increased Risk of Hypoxemia in Children infected with Influenza

**DOI:** 10.1093/ofid/ofaf695.1736

**Published:** 2026-01-11

**Authors:** Patricio L Acosta, F Martín Ferolla, Normando Mascardi, Pablo Neira, Agustina Chiormi, Mariana Reyero, Alejandra Retta, Maria Marta Contrini, Eduardo Lopez

**Affiliations:** Pediatric Infectious Disease Program, Hospital de Niños Ricardo Gutiérrez, Universidad de Buenos Aires, CONICET, Buenos Aires, Ciudad Autonoma de Buenos Aires, Argentina; Hospital de Niños Ricardo Gutierrez, Buenos Aires, Ciudad Autonoma de Buenos Aires, Argentina; Pediatric Infectious Diseases Program, Hospital de Niños "Dr. Ricardo Gutiérrez", Universidad de Buenos Aires, Buenos Aires, Ciudad Autonoma de Buenos Aires, Argentina; Hospital de Niños "Dr. Ricardo Gutiérrez", Universidad de Buenos Aires, Buenos Aires, Ciudad Autonoma de Buenos Aires, Argentina; Hospital de Niños "Dr. Ricardo Gutiérrez", Buenos Aires, Ciudad Autonoma de Buenos Aires, Argentina; Hospital de Niños Ricardo Gutiérrez, CABA, Ciudad Autonoma de Buenos Aires, Argentina; Hospital de Niños "Dr. Ricardo Gutiérrez", Buenos Aires, Ciudad Autonoma de Buenos Aires, Argentina; Hospital de Niños Ricardo Gutiérrez, CABA, Ciudad Autonoma de Buenos Aires, Argentina; Hospital de Niños Ricardo Gutierrez, Buenos Aires, Ciudad Autonoma de Buenos Aires, Argentina

## Abstract

**Background:**

Influenza remains a major cause of pediatric morbidity and mortality worldwide. Several factors can increase the risk of severe disease. *IFITM3* protein is a critical antiviral factor restricting influenza virus infection. The single nucleotide polymorphism (SNP) rs34481144 (C/T) in *IFITM3* has been associated with impaired immune responses, including reduced airway CD8⁺ T cells. We aimed to determine whether the rs34481144 variant is associated with influenza infection severity in children.

**Figure:**
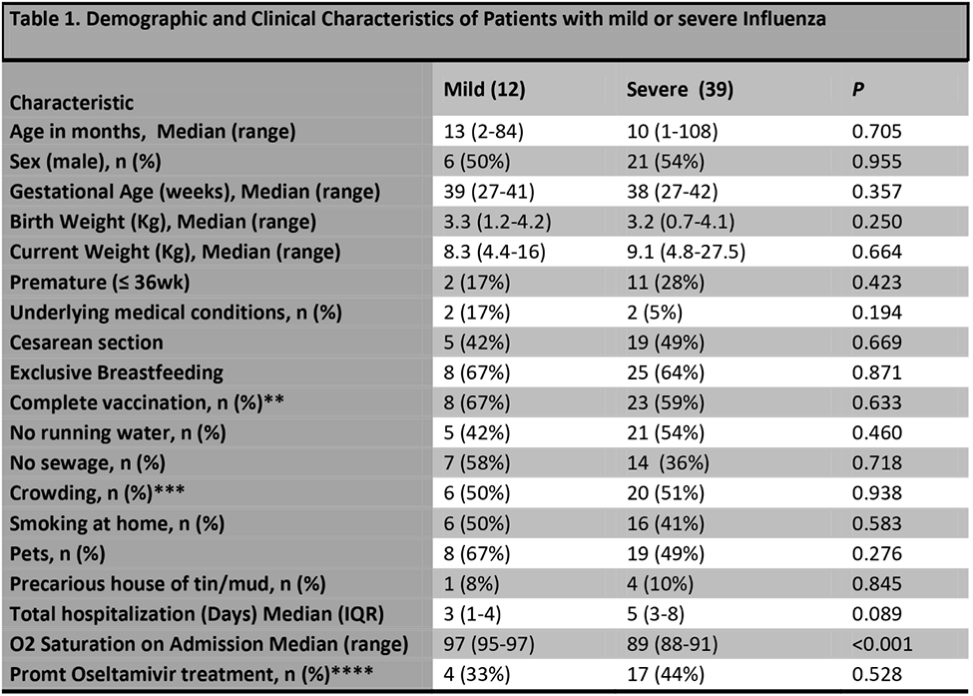
*Mild = not requiring supplemental oxygen therapy; Severe = requiring of supplemental oxygen therapy. **Immunization status, including Influenza vaccination (mandatory and free of charge for all patients <2 years old, or older with comorbidities, according to national immunization schedule) was also assessed. ***More than 3 persons per room. ****Within 48 hours of the onset of symptoms

**Figure:**
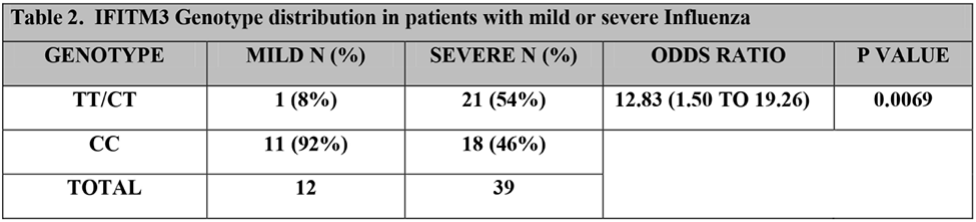
CC: homozygous wild type CT: heterozygous TT: homozygous for the alternative allele

**Methods:**

Prospective cohort study including children < 16 years old hospitalized with acute lower respiratory infection during 2019-2023 at the Ricardo Gutierrez Children´s Hospital. Patients who did not require supplemental oxygen therapy (SO_2_) were defined to have mild disease; severe cases were those who required SO_2_. Nasal aspirates were tested by RT-qPCR for RSV, rhinovirus, metapneumovirus, parainfluenza 3, influenza A & B. Viral quantification was estimated by RT-qPCR. DNA was isolated from samples for genotyping and *IFITM3* mutations were assessed by allelic discrimination with pre-optimized assays and performed by RT-qPCR.

**Results:**

401 children were enrolled. 51 (13%) were positive for influenza. Of them, 27 (53%) were male, the median age was 12 months and 4 (8%) had underlying illness. All were infected with influenza A type. Thirty-nine (76%) had severe disease. Characteristics of the population are shown in Table 1.

To determine whether SNP rs344811 was associated with Influenza disease severity, genotype was compared between mild and severe groups. The need of SO_2_ was increased among carriers of the alternative allele (rs34481144 T-genotype), with a significant detrimental effect *p*=0.0069; OR 12.83; CI 1.50 to 19.26 (Table 2). Furthermore, patients carrying the alternative allele had significantly lower oxygen saturation on admission (p=< 0.001). No significant differences were observed in viral load or sex distribution across genotypes.

**Conclusion:**

The *IFITM3* rs34481144 T allele is strongly associated with increased risk of severe influenza A infection in children. These findings highlight the importance of host genetic factors in the clinical course of Influenza and suggest a potential role for genetic screening in risk stratification.

**Disclosures:**

All Authors: No reported disclosures

